# Towards a Multi-level Exploration of Human and Computational Re-representation in Unified Cognitive Frameworks

**DOI:** 10.3389/fpsyg.2019.00940

**Published:** 2019-04-30

**Authors:** Ana-Maria Olteţeanu, Mikkel Schöttner, Arpit Bahety

**Affiliations:** Creative Cognitive Systems, Human-Centered Computing, Institute of Computer Science, Freie Universität Berlin, Berlin, Germany

**Keywords:** re-representation, cognitive systems, creative problem-solving, computational modeling, creativity

## Abstract

Re-representation is a critical ability to (i) understanding human creative problem solving, and (ii) modeling computational cognitive systems able to support or perform creative problem solving tasks on their own. This paper proposes a unified multi-level cognitive approach to investigating re-representation: the study of sensory-based, concept-based and problem template based possible forms of re-representations in an integrated manner. Descriptions and explanations of each level prepare the ground for further computational modeling. A study is deployed in order to explore the relationship between the various tasks proposed to reflect re-representation. A significant correlation between the investigated tasks is discovered. Two previous studies from the literature are replicated. A new strong and significant relationship between the Pattern Meanings Test and the Alternative Uses Test is observed.

## 1. Introduction

Imagine that you are given a problem, and try to solve it in vain for half an hour. After a while, you take a break from it and focus on something else. In the middle of this other activity, an altogether different way of seeing your previous problem pops into your mind. It feels like the elements of the problem have shifted, have reorganized in the mean time; that now you understand what is truly important, and how you should have tackled the problem in the first place. You experience a moment of clarity and promise: this new way of seeing the problem may allow you to find the solution, or at least to make further progress. It is quite possible you have, in the case above, experienced a case of re-representation.

Re-representation is a human cognitive ability encountered in processes of creativity and creative problem-solving. From a computational perspective, the better understanding and modeling of re-representation will enable us not only to study a cognitive skill to a deeper level, but also to implement the next generation of knowledge discovery and creative problem solving systems of cognitive inspiration. A computational system endowed with re-representation would have the same ability to view problems and datasets in different ways, generating new creative and insightful solution paths and discovering new knowledge. With re-representation being a cognitive process likely to become the next challenge in knowledge based systems, the main research questions addressed here are: (i) how can we better understand, characterize and empirically study re-representational abilities of the human mind, (ii) how can we begin to conceptualize and model them via computational modeling techniques? and (iii) is there promise that existing tasks from the empirical literature can be used to study re-representation in a cohesive manner?

Various cognitive abilities related to creativity have been studied in the literature, with computational systems capable of similar feats sometimes being implemented. Example of such abilities are: remote association (Mednick and Mednick, 1971; Olteţeanu and Falomir, 2015; Olteţeanu et al., 2017, 2018), analogy (Gentner, 1983; Falkenhainer et al., 1989; Holyoak and Thagard, 1996), metaphor (Lakoff and Johnson, 1980, 1999; Veale and Hao, 2008), concept blending (Fauconnier and Turner, 1998; Eppe et al., 2015), etc. Being part of the creativity cognitive skills family, an ability for re-representation might (i) be related or similar to some creativity skills (e.g., analogy and metaphor), or (ii) use other skills on which the creative process relies on (e.g., association). However, before making any claims regarding relationships to other creativity cognitive skills or processes, a stronger systematic approach to generally understanding, characterizing and studying re-representation is necessary.

Various cognitive architectures with different types of theoretical and representational commitments exist, being used for the modeling of various sets of tasks and processes: ACT-R (Lovett, 1998), SOAR (Laird et al., 1987), CLARION (Sun, 2007), EPIC (Kieras and Meyer, 1997), Leabra (O'Reilly, 1996), SAL (Jilk et al., 2008), etc. However, no such architecture deals with creative processes in a unified manner. Unified approaches to some processes have been proposed (Olteţeanu, 2014; Olteţeanu, 2016), but they do not yet involve re-representation.

To start an unification in this direction, re-representation as a process needs to be studied in a variety of different settings, so that the cognitive framework accommodating one facet of the process can also deal with its other manifestations. In the unified spirit of cognitive architectures put forward by Newell (1994), this paper proposes that cognitive frameworks aimed at studying and implementing re-representation would need to systematically do so at different levels of cognitive skill. This would require defining different levels which have chances to be unified by the same process, and doing so in terms which make the process amenable to computational implementation. Here, an approach in which multiple possible levels of re-representation are explored is proposed, and these levels are discussed in computational modeling terms. The main aim is to set a foundation toward the combined empirical and computationally sound study of re-representation in cognitive architectures. These levels are related to specific existing creativity tasks, to offer a good shot at empirical falsification of this unified re-representation study hypothesis, and a sturdy benchmark to compare future computational models against. An exploratory study is deployed to confirm or refute initial relationships between these tasks.

The rest of this paper is organized as follows: the three levels of re-representation are first described in section 2. The first level—of *feature to object re-representation*—is explored in possible computational and modeling terms in section 3. The second level—of *object and properties re-representation*—is described in section 4. The third level—of *objects and problem template re-representations*—is explored in section 5. The study deployed to explore the relationship between these tasks is reported in section 6. An overview of the possible similarities, differences and interplay between the various levels is discussed in section 8, together with the study results.

## 2. Three Levels of Re-representation Described Through Various Creativity Tasks

In the following, four different creative problem solving tasks will be briefly described, together with their possible relations to the ability for re-representation. The tasks are:

1. the ambiguous figures task;
2. the pattern and line meanings tests (Wallach and Kogan, 1965);
3. the Alternative Uses Test (Guilford et al., 1978) and
4. insight problems (Maier, 1931; Duncker, 1945; Dow and Mayer, 2004).

Based on these four tasks, three potential levels of re-representation will be discussed.

**Figure 1:**
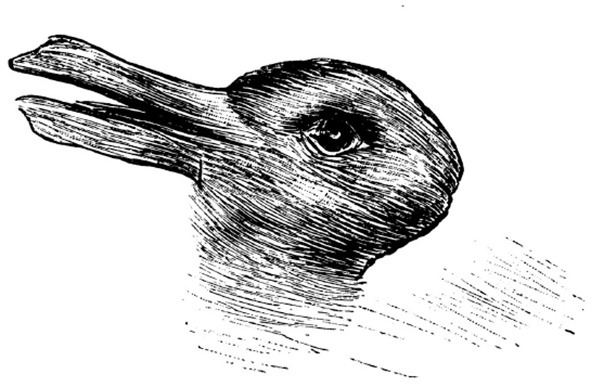
The duck rabbit illusion.

Imagine you are looking at an **ambiguous figure**, like the one shown in Figure 1, and can only initially see a rabbit. You might be told that a different image (a duck) can also be seen, and voluntarily attempt to see it, or you might experience a spontaneous surprising moment in which the second image “pops up” all of a sudden. Now you can most likely switch between seeing these different images at will. In fact, participants presented with this task might be asked to press a button every time they are able to switch their representation of the stimulus (Doherty and Mair, 2012)—this evaluative measurement is called *frequency of reversal*. How are ambiguous figures related to re-representation? Our approach proposes that ambiguous images can stand in for a level of the ability which involves level of an ability for re-representing visual features in images as different possible objects or entities.

**Figure 2:**
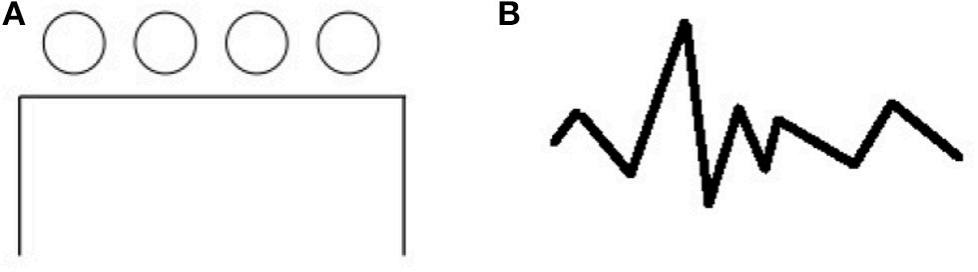
Creativity tests by Wallach and Kogan (1965). **(A)** pattern meanings test. **(B)** Line meanings test.

The **pattern meanings test** by Wallach and Kogan (1965) gives participants abstract pattern stimuli like the one shown in Figure 2A. The task of the participants is to describe, verbally or in writing, all the things that they think these patterns could be, or all of the things that these patterns remind them of (note the reference to memory, which we will pick up again in section 3.2). For example, the pattern in Figure 2A can be seen as a collection of barstools in front of a table, people waiting in line next to a building, flower heads coming from a rectangular flower pot, a Newton's cradle toy etc.

A similar task is presented by the **line meanings test** (Wallach and Kogan, 1965), in which a line like the one in Figure 2B is presented and the participants have to come up with things the line could be—for example the contour of a mountain chain, an uneven saw, a comb with broken teeth, etc^1^.

The pattern meaning and line meaning tests have in common the following: during both tasks, an abstract stimulus (or set of features) is repeatedly re-represented by the participants through various known representations.

The number of alternate interpretations that participants come up with is part of the evaluation of results in pattern and line meaning tests. This type of interpretation and re-representation can still be positioned at the level of interpreting features as objects. Unlike in the ambiguous figures task, the objects through which the re-representation is made are not restricted to two, nor present in the figure; they rely on the knowledge of the solver. Because of this, more than two interpretations are also possible (ambiguous figures can generally be represented in two, or a maximum of three ways).

The **Alternative Uses test** by Guilford et al. (1978) gives participants an object, and asks them to come up with as many uses as they can for it. For example, the object “brick” is given, and participants can come up with uses like the following: a brick can be used as a paperweight, a weapon, a doorstop, around the fire, to write on the pavement with, etc.

In order to connect this task to re-representation, the process of coming up with different possible uses needs to be considered. A proposal for how this type of creative inference (coming up with alternative uses for objects) can be done is the following: after the object has been given (e.g., dental floss), its properties (shape, weight, material, size) and their combinations are re-represented as other possible objects (the shape and material of *dental floss* make this object easy to re-represent as a *long piece of string*, for example). Then, the affordances of the objects they were re-represented into can be applied back to the initial objects (for example, dental floss can be used for sewing). This very inference is actually part of the set of creative inferences made by a system computationally implementing this process (Olteţeanu and Falomir, 2016).

This is thus a case for re-representation if the properties or features of an object are interpreted as properties and features of another object, in order to come up with the new affordance. Therefore, the Alternative Uses test might involve the level of an ability to re-represent objects and their properties as different objects in a household domain environment, in order to be able to come up with creative uses for said objects. This level of re-representation, together with the process by which OROC (Olteţeanu and Falomir, 2016), a prototype computational system capable of performing this task, produces answers, will be discussed in more computational detail in Section 4.

**Figure 3:**
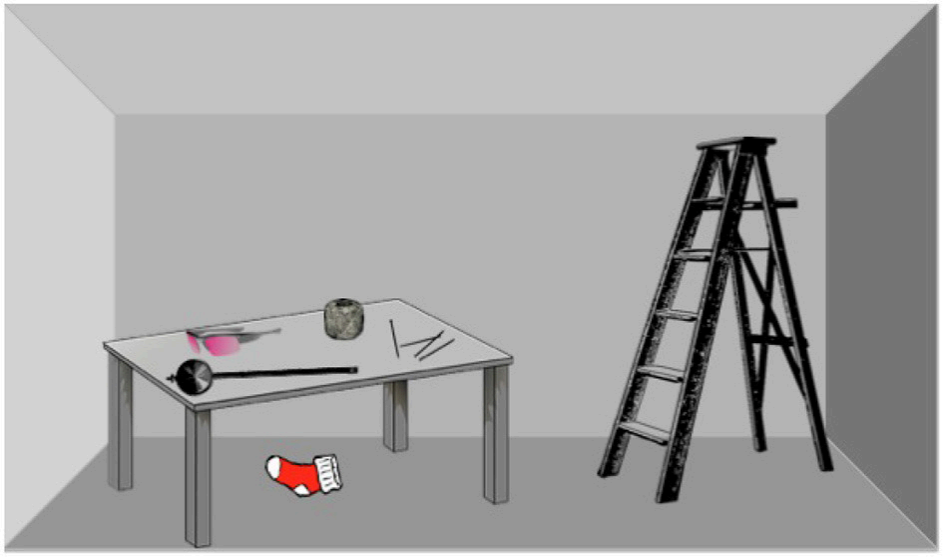
The weight problem.

**Insight problems** represent more complex tasks, in which a set of objects (daily objects, abstract objects or sometimes just patterns) in various relationships are presented to the solver. For example, the weight problem (Duncker, 1945), which we illustrated by Figure 3, is stated as follows: *You are to help the observer set up the room for an experiment. You need to attach the pendulum to the ceiling. What do you do?*

The participants start thinking of various action plans (involving sets of actions, combination of objects and object affordances) that can help them solve the problem. After a while, it becomes clear that one of the core issues of solving the problem is the ability to attach one of the nails on the table to the ceiling, in order to fix the string on the ceiling and attach the pendulum to it. Various ways of achieving this are attempted. A productive path is the endeavor to create a hammer out of existing objects—attempts to realize this involve human solvers proposing to use the top of the staircase as a hammer, wrapping the spool of string or folded plastic glasses in a sock in order to make them “harder,” or, Duncker's correct solution, using the pendulum itself to hammer in the nail before attaching it to a piece of string. This shows re-representation at work in insight problems—re-representing some objects (the pendulum) as another needed object (the hammer)—in a flavor which is similar to OROC's processes when solving the AUT. However, multiple objects and sets of actions might also be represented in multiple ways, using already known ways of solving problems (what we call problem templates) and their subsets. A deeper examination and example of this level are shown in section 5.

In summary, the three levels at which re-representation can be explored proposed here are:

1. the ability to re-represent features as different sets of objects or images in the case of ambiguous figures;
2. the ability to re-represent objects and their properties as different objects; we will generally discuss this in a household domain environment, focusing on the ability to use objects creatively when traditional objects that would be used are missing^2^ and
3. the ability to re-represent objects and scenes under different problem templates, as to be able to computationally tackle the solving of human insight problems in the future.

**Figure 4:**
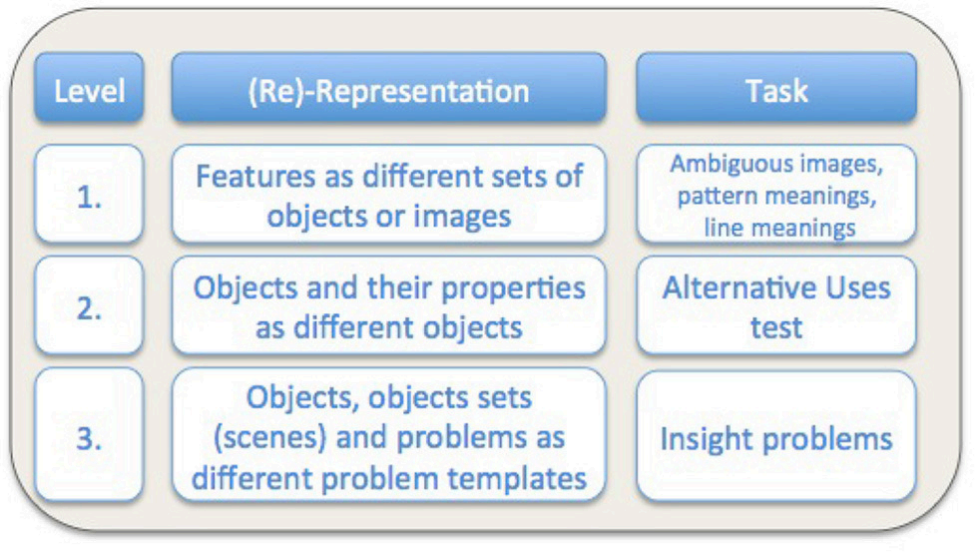
Overview of the proposed levels of re-representation.

An overview of the various levels of re-representation and empirical tasks with which re-representation could be studied at that level is presented in Figure 4. This is of course meant as the initial sketch, not an exhaustive list.

Some preliminary evidence points in the direction of the validity of such an approach: various theories have anticipated the relation between ambiguous figures and creativity, the earliest being expressed in the gestaltist movement (a nice summary of the general gestaltist views on restructuring can be found in Ohlsson (1983, 1984)). Recently, the first empirical investigations to show results in confirming this direction of thought have been obtained by Wiseman et al. (2011) and Doherty and Mair (2012). In Wiseman et al. (2011), a correlation between ease of ambiguous figures reversal and creativity as measured using Guilford's Alternative Uses Task has been observed (Spearman's *rho* = 0.28, *p* = 0.007). Doherty and Mair (2012) have shown a correlation between ambiguous figures and creativity as measured using the pattern meanings task (Spearman's *rho* = 0.42, *p* < 0.01).

### 2.1. Setting up the Approach

In the following, each level of re-representation will be analyzed in computational terms, using the context of the tasks of which an overview was provided in section 2. The levels will thus be defined in terms of input, pre-existing knowledge in the cognitive system, output, and processes of re-representation.

These are described below:

1. the **input** – defines the initial sensory or conceptual input of the task (in objective terms), be it features, objects, their properties, object groups, scenes or problems, or descriptions thereof;
2. the **pre-existing knowledge** – estimates what kind of knowledge needs to be assumed to exist in the system, in order for the processes of re-representation and various types of output to be possible;
3. the **output** – represents the types of images, objects, uses, object groups and plans of action (or representations thereof) that are the results of the problem representation and re-representation process;
4. **re-representation** – the process which we are trying to observe at these various levels, takes into account existing knowledge and input, as to produce various forms of output.

The following sections (3–5) explore how each of the previously sketched levels of re-representation can be described in such computational modeling terms.

## 3. Level 1—Features Re-represented as Different Objects and Images

Level one refers to the ability to re-represent features as different objects and images. The ambiguous figures tasks is tackled in section 3.1. The pattern and line meaning tasks are explored together in section 3.2. Section 3.3 provides a short summary of the analytically observed traits of re-representation at this level.

### 3.1. Ambiguous Figures

The objective input in the duck-rabbit figure can be taken as a set of visual features. The output is two images (or image interpretations). The pre-existing knowledge is the knowledge of the object represented in the image, or sometimes familiarity with such a type of representation of this object^3^.

**Figure 5:**
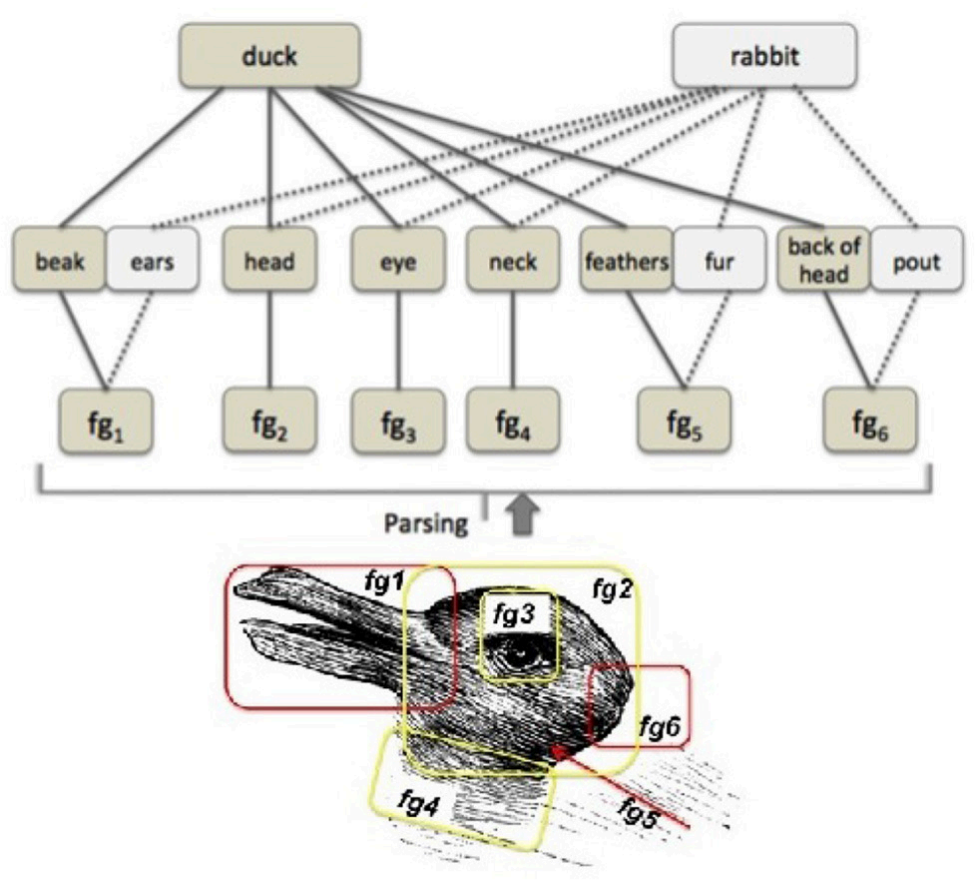
The duck-rabbit ambiguous figure. (Bottom) Split in feature groups. (Top) With feature groups (*fg*_*x*_, *x*∈{1, 2, …, 6}) parsed as object (animal) parts.

In order to interpret such features as being part of and representing an object, a subset of these features has to be selected, grouped and matched to a known object or image. Some such sets of features can be pre-grouped in feature groups which can be mapped to various object parts, as shown in Figure 5 (bottom). In this case, the various feature groups are matched to parts of the body of the animal which is seen as output. However, various feature groups can be interpreted as different body parts, as shown in Figure 5 (middle). Thus, whether one sees the duck or the rabbit can depend on which parts these feature groups are interpreted to be.

In the case shown in Figure 5, interpreting feature group *fg*_1_ as a *beak* or *ears*, *fg*_5_ as a *feathers* or *fur* texture and *fg*_6_ as the *back of the head* or the *pout* might make all the difference to seeing one of the images, or the other. It is unclear in which order the process happens: it might be that (i) cognitive systems directly cast features in terms of one interpretation or the other (a top down view), or that (ii) the various sets of features trigger the body parts, which then trigger the interpretation of the image (a bottom up view) or that (iii) one of the features, on which the system is focusing, triggers the bigger picture, which in turn triggers the interpretation of the body parts (a mixed view)^4^.

However, it is clear that (images of) ducks and rabbits must have been encountered previously. The ability to *switch* between these representations is the ability to (a) switch between a potential mapping of the (objective) features to different feature groups and/or objects or (b) switch between different interpretations of these feature groups as objects. In the case of the duck-rabbit illusion, a switch might very well be influenced by (b) - the ability to re-categorize one of the initial sets of feature groups as a different animal body part. However, in the case of different ambiguous figures, where feature groups cannot be allocated very neatly to one or another interpretation, features groups themselves might need a different re-grouping to be able to map to (or under the mapping of) different body parts. Thus (c) the ability to switch between different objects as possible interpretations of features and thus arrange them in feature groups might come into play. All three switches are different facets of the re-representation process.

**Figure 6:**
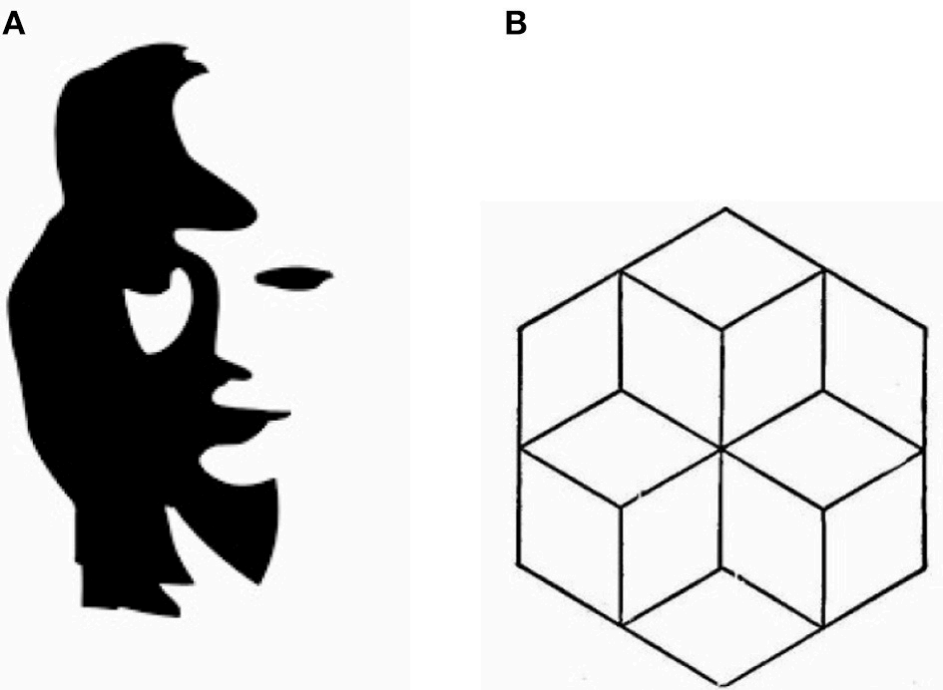
Different illusions. **(A)** The girl saxophonist. **(B)** A three interpretations ambiguous figure.

Before moving on to the pattern and line meanings task, it is worth noting that not all ambiguous features are about a re-representation of visual features as different groups or an interpretation of such groups as different categories. For example, in the girl-saxophonist illusion (Figure 6A), the relations between feature groups are not one to one between the two images. The switch between the two representations in such cases seems to be done apart of the level of interpreting the feature groups as different parts, at the level of choosing what features to group to start with. Thus, grouping the features required to categorize the saxophone already precludes grouping the girl's nose, chin and their shadows, while grouping the girl's mouth precludes part of the saxophonist's coat. This also appears in insight problems, where seeing one object with a particular affordance might impede solutions which include the same object having different affordances.

Furthermore, sometimes such relations are not between visual features groups, but rather between interpretations of spatial relations. For example, Figure 6B shows an image with three possible interpretations. The switch between two of these—whether the three cubes are perceived as forming a pile on the floor or hanging from the ceiling—depends on whether certain surfaces are interpreted to be above or under other surfaces, and whether they are emphasized as part of a certain cube or another. If the shapes are perceived without projecting depth relations over them, a different (2-D) image is perceived.

### 3.2. Pattern and Line Meanings

In the pattern and line meanings tasks, the input is still a set of features. The output consists of different objects, groups of objects and potentially scenes (e.g., a people waiting in line representation of Figure 2A) as an interpretation. The pre-existing knowledge is knowledge about said objects, groups of objects, contexts, relations and scenes, which is then brought to bear on the interpretation.

A re-representational overlap can again be noticed: different objects can all be projected on the initial set of features. The pattern and line meanings task present a variant of the representation and re-representation of features level, in the sense that multiple interpretations can be made, and the set of features can be matched to a much wider set of visual depictions. Indeed, if ambiguous figures generally accommodate a maximum of three possible interpretations, pattern and line meanings can be interpreted as a set which is only restricted by featural similarities to the initially presented stimulus. Thus pattern and line stimuli from such tasks are much more ambiguous than ambiguous figures (despite the name of the task), if the measure of ambiguity is how many mappings can be made from the stimulus to known objects or scenes representations.

The figure stimuli are also much more abstract than the ones presented in the ambiguous figures task, and many more features need to be added to construct a full representation. Ambiguous figures can be considered as “abstract” stimuli as well of course, in the sense that they are not complete 2D depictions of the object (like a full image), but rather drawings or sketches. However, the sets of features in the ambiguous figures contain much more information about the figure being represented, thus constituting a much more complete representation.

Ambiguous figures rely on the identification of an already known pattern, which pre-exists in the memory of the system (or has been experienced in a similar form), with the main task being one of discriminating which features to take into account and in which role. The pattern and line meanings task rely much heavier on memory, as multiple known similar patterns can be elicited, and, in a sense, the more such patterns a system has acquired, the higher its potential performance in such a test, if re-representational abilities are in place (thus if the participant can switch with ease from one representation of the initial feature set to another). The same can be said about the line meanings test (Wallach and Kogan, 1965), with the only difference that, while the line meaning test has to be interpreted without interrupting the line, the mapping in the pattern meanings test might be from multiple parts of the feature set to multiple objects. These objects might have a common context: for example interpreting the four circles in Figure 2A as flower heads contextually constrains the interpretation of the incomplete rectangle as a flower pot, while interpreting the same circles as bar stools or chairs contextually constrains the interpretation of the incomplete rectangle as a bar or a table.

Though the objects and scenes to which the pattern matching is being done have to already exist in the knowledge base of the cognitive system solving the task, a look at some of the answers given by people when attempting this task shows exactly how diverse and multi-faceted the human ability for re-representation is.

The interpretations provided for Figure 2A show, amongst other things, an ability to look at and imagine scenes from various perspectives: for example, the barstools in front of a table are viewed from above; the flower heads coming from a rectangular flower pot are viewed from in front and possibly from the same height, the Newton's cradle toy is viewed upside down (and only has four balls), etc.

Another interesting example of re-representation in the pattern meanings task is depicted in Table 1. Here, the pattern given as the stimulus, together with a subset of 11 answers given by a human participant have been depicted visually. As the responses have been given verbally, this visual depiction might not be exactly what the participant had in mind, but we attempted it in order to showcase the visual similarity of answers to the pattern stimulus. As can be seen in Table 1, the answers are quite varied and the similarity is very flexible. The representational poses of the objects to which the similarity comparison is done are also quite varied and flexible.

**Table 1:**
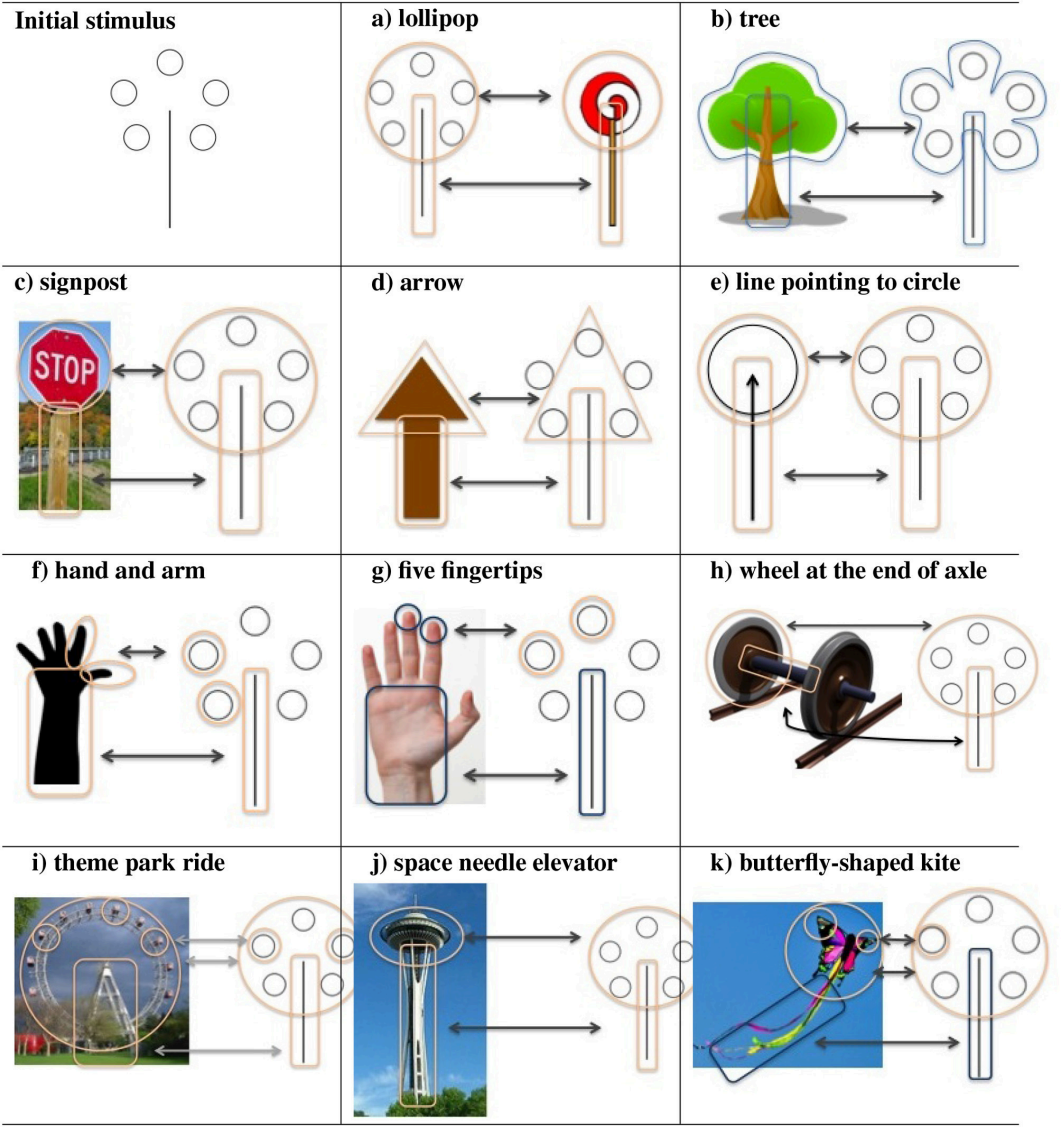
Series of answers by a participant to a pattern meaning stimulus, their visual depiction and types of similarity with stimulus emphasized.

Procedures of interpretation of the various parts of the stimuli as a group are at play from the very beginning. We also see Gestaltic procedures of completion—thus the five circles of the initial stimulus are interpreted as representing a circle in a variety of answers, like in (a),(c),(e), etc. We observe procedures such as tilting of the various objects—like the circle in (h), the *wheel and axle* answer, or the stem in (k), the *butterfly-shaped kite* answer. We observe contour shape modifications: for example, the circle in the initial stimulus has to be compressed to an oval to match (j), the *space needle elevator*. Other types of pattern completion do exist: for example, it is the contour of the 5 circles that seems to determine answers (b) and (k). Only three such circles are selected for the group required in answer (d) *arrow*, and the pattern completion is done to the shape of a triangle—the three circles form its vertices. The number might play a role in answers (f) *hand and arm* and (g) *five fingertips*. Answer (i) *theme park ride* contains both a mapping of the initial circles and of the imaginary completion one, whilst possibly (f), but more clearly (g) map the initial circles too.

These examples speak about a process of parsing and re-parsing initial features, while using them to search through a variety of known objects, in multiple inclinations and poses. Besides being answers, these can also be interpreted as visual (imaginary) comparisons of known objects to existing features of the stimulus, while searching for possible matches.

Also, various types of similarity of matches can exist, depending on which visual properties are involved—for example, a set of shape contour features initially taken from a mushroom can be mapped to an icecream shape, however a different set (e.g., the striations under a mushroom's cap) could trigger the possibility of re-representing the mushroom as an umbrella. Thus parts of the visual pattern might trigger certain possible interpretations, while other features might trigger others^5^.

### 3.3. Summary—Level 1

The first level refers to the interpretation of grouped or ungrouped sets of features as possible known images, objects or scenes. The input in this case is features. The output is images, like in the ambiguous figures task, and objects or scenes, like in the pattern meanings and line meanings task. These objects need to have been previously known, though the particular amount of knowledge required by each task can be different.

The process of re-representation is, in this case, the process of mapping a set of input features to different types of known objects and scenes, and switching between such various mappings. This output is interpreted as being represented, in an incomplete form, in the input: as sketches (ambiguous figures), or as allusions to an object or a set of objects/visual scene (in the line meanings and pattern meanings tasks).

## 4. Level 2—Objects and Properties Re-represented as Different Objects

Level two refers to the ability of using existing objects as if they were other objects (*A newspaper can be used as an umbrella* or *A cup can be used as a bell*) and coming up with creative uses for existing objects (like *A wooly shoe can be used to wipe kitchen surfaces with*). Some such forms of creative inference are imperfect: some properties and/or some object parts might be missing from the initial object, in order for it to be properly transformed into or re-represented as a different object. An example of properties missing: a magazine, made of glossy paper, and preferably of a large size, might work much better as an umbrella than a newspaper; some of the newspaper's properties (being made of paper) come slightly short of getting the job done as an umbrella. As for missing parts, a cup is not yet a bell, it still requires other parts, perhaps a spoon to hit it with or some other form of clapper^6^.

The input to level two is objects and their properties. The output is (a) other objects and/or (b) new affordances for existing objects in the specific case of the Alternative Uses test.

The output is other objects (case a), when the re-representation is done overtly, referring to objects: *I can use a shoe if I don't have a hammer, to hammer in the nail*. The *shoe* and the *hammer* are already known, while the creative inference might be known or made on the spot. If re-representation is indeed at play at this level, the assertion *A cup can be used as a bell* can be taken as *I can re-represent the shape and material features of a cup as the body of a bell*. In a more general case, assertions of the form *I can use object* *A* *to replace object* *B, in the context of task* *X* can be taken as *I can re-represent (a subset of) the features and properties of object* *B, or the features and properties required for task* *X, as object* *A*.

The output can be an affordance (case b), like in the case of the Alternative Uses test, when the answer given by a participant is that a brick can be used to hit a nail, driving it through the wall. A covert reference to known objects which one has performed this task with (like a hammer, or a stone) might be made in the process. The initial object, the covert referent object and its affordances must all be known for the creative inference to be made. Answers that *A brick can be used to hit a nail* can be taken as *I can re-represent (a subset of) the features and properties of a hammer, which has the affordance of putting a nail in the wall, as a brick*. More generally, answers of the type *I can use object A for affordance X*, can be taken as *I can re-represent (a subset of) the features and properties of object B, or features and properties connected to affordance X, as object A*.

A small set of answers to the Alternative Uses test question ‘*What can you use a cup for?'* is shown in Table 2. The third column shows overt and possible covert re-representation objects. As mentioned above, this might be done with or without covert object references; if done without, such inferences can be based on existing sets of properties (from previous objects) which co-occur with the performance of a particular task.

**Table 2:** Alternative Uses test example, showing answers elicited and possible re-representations of the object *Cup*.

**Object**	**Answers**	**Object re-representation**
Cup	Draw a circle	Circle form
	Boil things in	Pot
	Keep door open	Doorstop
	Hinder something falling	Bowl
	Home decoration, if on a nail	Decoration
	Smash it for sharp edges	Shards
	As a hat	Hat
	Cap for a bottle	Bottle cap
	As a mace to smash garlic with	Mace
	To make sand castles	Sand bucket
	To dig earth	Shovel

**Figure 7:**
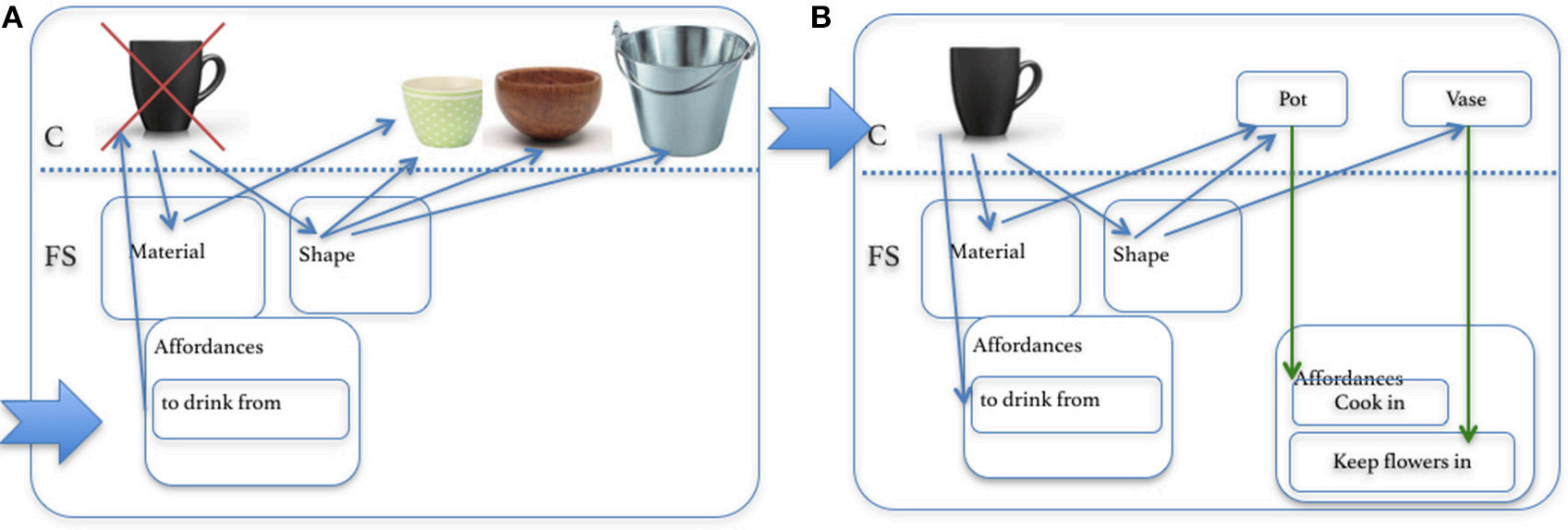
Using re-representation of properties for **(A)** object replacement and **(B)** alternative use answers in OROC. The process follows the direction of the arrows.

An example of an implementation of these principles is the OROC system (Olteţeanu and Falomir, 2016), which uses knowledge of object material, shape and other properties to make both object replacements and inferences in the style of the AUT, showing that the same core process can be applied to both types of tasks. Figure 7A shows the case in which OROC needs to find an object replacement for a *Cup*. OROC has previously encoded a *Cup* with a set of material, shape, size, affordance and other properties. It then uses its type of knowledge organization to search for other objects which it has encoded in properties of the required object—thus finding and proposing for the task objects like a *Bowl*, *Vase* and a *Bucket*, which have similar material and shape.

Similarly, with a small extension of this process, OROC proposes affordances to a particular object, thus being able to answer AUT questions. Figure 7B shows this process. Objects with which the initial object shares (similar) properties like shape and material have affordances which are transferred to the initial object. This can be interpreted as a process of re-representation of an object (or a subset of its properties) through knowledge and properties pertaining to another object. A *Cup* is thus re-represented as a *Pot*, *Bowl* or *Vase*, and then the affordances of such objects are attached to *Cup* as creative inferences of possible uses. OROC uses similar objects for creative object composition.

Thus, in the case of this level, what is being re-represented is an object, or sets of object parts, features and properties, as different objects. It might be that such properties are already memorized and/or organized in the memory of the solver or creative agent, or that the features of the given object are re-represented in order to produce new uses *ad hoc*.

## 5. Level 3—Objects and Problem Re-representation

In order to showcase re-representation at the level of insight problems, the case of such an insight problem, a problem involving practical objects, will be analyzed. Practical object use insight problems make for a worthy case study for the obvious reason that re-representation(s) in such a problem is much easier to talk about in concrete terms (about objects, actions, plans and affordances) than in abstract insight problems (which might involve abstract re-representations).

In the case of practical object insight problems, the input is a visual scene—that is a set of object or object groups, in various relations to each other^7^. The output is an action plan of how to solve a particular problem—that is a set of actions using objects, and putting objects in various relations in the process^8^. This requires existing knowledge on objects, object properties, parts and relations, actions with objects and consequences of such actions. We call knowledge about sets of objects, actions involving them and their relations, and the consequence of these actions *problem templates* (Olteţeanu, 2014). Such problem templates might have been learned while interacting with objects, or through communication. The consequences of particular known problem templates may or may not be helpful in coming up with the solution to a particular current problem.

**Figure 8:**
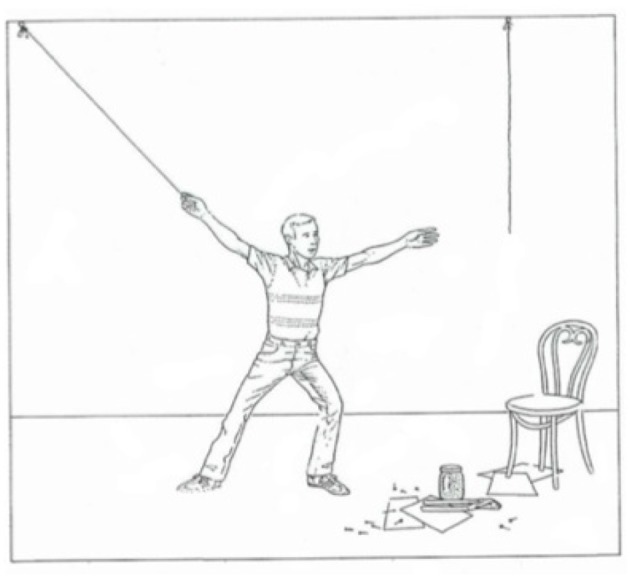
The two strings problem.

In the process of solving an insight problem, re-representation involves seeing the problem in a different way, that is recasting the problem in different problem templates, object templates or other pre-existing representations. These terms will become clearer as we take a look at the two strings problem. This problem is presented as shown in Figure 8. The participant is told: *A person is put in a room that has two strings hanging from the ceiling. They have to tie the two strings together. How can they do this, knowing that it is impossible to reach one string while holding the other?*

**Figure 9:**
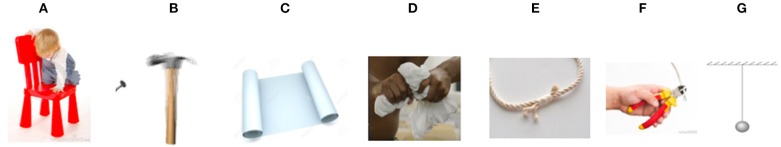
Visual depiction of different representations used to organize or re-represent problem objects when solving two strings problem—from **(A)** hammering a nail in the wall to **(G)** pendulum.

While attempting to solve this problem, human participants come up with different possible fragments of the solution (as shown by a think aloud experiment) or with constructions which point to a process of re-representation of the problem objects through various problem and action templates, or object templates. For example, some people try to build solutions which involve:

- climbing on the chair. This requires grouping existing object elements into a small problem or action template like the one shown in Figure 9A, which has as a consequence the person in the problem being in a higher position; some participants think that being higher will allow them to reach further by grabbing one of the strings at a different point;
- hammering the ropes to the ceiling (this is afforded by first climbing on the chair). This involves the re-representation of elements of the problems like the *nail* and the *pliers* via existing knowledge or representation pieces, like the one shown in Figure 9B; in the process the pliers are also re-represented as a hammer;
- rolling *paper*, as shown in Figure 9C—this is useful when the paper itself is re-represented as a string extension or a string connector, like in Figure 9E;
- rolling the T-*shirt* of the person in the room into a piece of rope, as shown in Figure 9D. This again allows re-representation and use of the T-*shirt* object as a form of a rope object;
- connecting strings or items that can be re-represented as a string (*paper*, T-*shirt*), like in Figure 9E, sometimes with the purpose of prolonging them or the initial two strings;
- using the *pliers* to grab one of the *strings* hanging from the ceiling, thus possibly using a pliers and wire representation like the one in Figure 9F to re-represent existing pliers and wire-like objects (e.g., the strings); in the context of this problem, using the *pliers* to grab the *string* also has the consequence of elongating one's arm reach;
- or, the actual acknowledged solution to this problem—building a pendulum like the one in Figure 9G. This is done by using a string and a heavy object in the room (like the *pliers*), attaching the heavy object to one of the strings, and using its new affordance as a pendulum to swing it around, having it move toward you while you are holding on to the first string. This requires recasting or re-representing the *string* and *pliers* or the *string* and *jar* objects and/or their properties through the template of a pendulum. Thus, an object template is used to re-represent part of the problem objects, leading to a new type of affordance, and a possible solution.

Some such problem solving fragments might involve simple grouping of existing elements (like Figure 9A) in an existing representation, and are straightforward. Others might involve more complex forms of re-representation, in which various objects are replaced with others—like nailing the rope to the ceiling with a non-existent hammer (Figure 9B)—which has to be re-represented from another object (pliers) or its set of features.

Furthermore, the use of various templates allows for others to be deployed, as shown in the previous examples: climbing on the chair is a necessary part of hammering the ropes to the ceiling; rolling the paper or the cloth is necessary in deploying another template which involves lengthening and connecting the ropes, or whatever objects are now re-represented as the ropes, etc. *Some object groupings and higher level representations of sets of objects as one functional unit might thus trigger other objects in the scene to be grouped in a certain way, in the same manner in which interpreting a set of visual features as part of a certain group might trigger or be related to already existing possible interpretations of the entire image*^9^.

The use of certain representations precludes or at least impedes others being used. For example, if the *pliers* have been re-represented as a hammer and part of a *fixing the string to the ceiling with a nail* problem template, human solvers might encounter difficulties in re-representing (the same pliers) as a weight, part of a *pendulum* template. This is similar to what we remarked in the case of some ambiguous figures—when one set of features has been represented as a nose, they or an overlapping subset cannot be represented as a saxophone at the same time. Re-representation thus is required here too, just at a different level.

To explore whether relations between these tasks can be found empirically, and thus re-representation can be studied at multiple levels, a study involving these tasks was deployed and is reported in the following section.

## 6. Study

### 6.1. Participants

A sample of 175 participants was recruited using Crowdflower, 37 of which had to be excluded due to giving incomplete or nonsensical answers. This resulted in a total sample size of 138 participants (63% females, 37% males) whose answers were used for further analysis. Ages were recorded using an ordinal scale with age brackets of 10 years. The age ranges of the participants were: under 20 years (*n* = 8), 20–30 years (*n* = 30), 30–40 years (*n* = 49), 40–50 years (*n* = 23), 50–60 years (*n* = 24), 60–70 years (*n* = 3), and over 70 years (*n* = 1). Most participants had higher education with 58 (42%) having completed undergraduate courses and 16 (11.6%) having completed postgraduate courses. Nine (6.5%) were enrolled in postgraduate courses, 12 (8.7%) in undergraduate courses, 31 (22.5%) had finished their high school diploma, and 12 (8.7%) finished secondary school. Using a 5-point Likert-scale, self-ratings of both creativity (*mean* = 2.81, *SD* = 1.04) and problem-solving (*mean* = 2.88, *SD* = 0.96) were gathered.

An approval by the ethics committee was not necessary as per our institution and national guidelines, because no risks were attached to the study. Informed online consent was given by the participants to use the anonymized data for research purposes. Participants received a payment in Crowdflower for their participation.

### 6.2. Method

#### 6.2.1. Ambiguous Figures Task

For this task, five different pictures showing ambiguous figures were used. Participants were first shown the picture and asked what they saw. Following that, they were informed about the two different figures that can be seen in the picture. They were asked if they could see both figures. Participants were then shown each picture for 1 min, while they were instructed to mentally switch between figures and press a button whenever they could see the other figure. Both the number of key presses and the time passed between presses were recorded.

#### 6.2.2. Pattern Meanings Test

In the pattern meanings test, participants were shown six different patterns composed of simple abstract geometric objects, one at a time. They were instructed to write down “as many things as they could come up with for what the object can be.” The time the participant took for each pattern and his or her answers were recorded.

#### 6.2.3. Alternative Uses Task

Participants were given an everyday object, to which they should give as many alternative uses as possible. This procedure was repeated for 5 different objects. Those objects were *carpet, newspaper, cup, dental floss*, and *toothbrush*. Again, the answers and how much time participants took for each object were recorded.

#### 6.2.4. Insight Tasks

Two insight problems were given to the participants: the candle problem and the Jack and Jill weight problem . They were instructed that these problems could have several possible answers and that they should think of possible solutions and write them down. The problems were formulated as follows:

“*You are given a candle, a box of thumbtacks and a book of matches. You are supposed to fix the lit candle unto the wall in a way that does not allow the wax to drip below. What do you do?*” (candle problem).

“*Jack and Jill are arguing about who weighs more. What could they do to find out for certain?*” (Jack and Jill weight problem).

Each of the problems was accompanied by a picture, both of which can be found in the Appendix, together with the image stimuli of the other tasks. The tasks were always given in the same order. However, the stimuli in each category were presented in a randomized manner.

### 6.3. Results

#### 6.3.1. Ambiguous Figures Task

The results for the ambiguous figures task can be seen in Table 3, which shows the mean number of switches per minute for each figure, as well as an average over all trials. Additionally, standard deviations are shown. Mean time until switch tells how much time passed on average until a switch between the two representations was reported. For example, for picture 1, participants produced on average 19.64 key presses (*SD* = 21.12), and were able to switch between representations about every 3 seconds.

**Table 3:** Results of the ambiguous figures task.

	**Picture 1**	**Picture 2**	**Picture 3**	**Picture 4**	**Picture 5**	**Overall**
Mean key presses	19.64	27.58	25.08	31.56	34.32	27.63
SD	21.12	22.31	23.45	23.91	28.72	24.51
Mean time until switch in seconds	3.05	2.18	2.39	1.90	1.73	2.17

Additionally, the number of key presses over time for all participants was plotted using frequency polygons. This was done for pictures 1–5 individually and for all pictures combined. These graphs can be seen in Table 4.

**Table 4:**
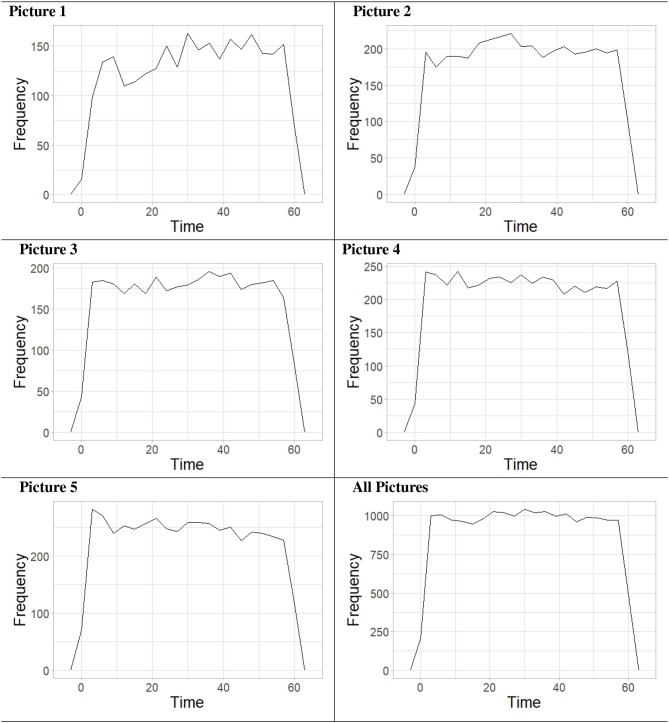
Frequency of key presses over time for each of the pictures used in the ambiguous figures task, as well as for all of the pictures combined, displayed in a frequency polygon.

*The x-axis shows the time in seconds and the y-axis the number of key presses*.

#### 6.3.2. Pattern Meanings Test

Results for the pattern meanings test are shown in Table 5. *Fluency* is the number of answers given; *Flexibility* measures how many semantic domains these answers cover, based on human ratings. Additionally, *response times per pattern* (the time a participant took for giving all the answers), and *response times per meaning* are shown. The response times per meaning were not recorded directly—they are averaged from the number of responses produced and the total response time per pattern.

**Table 5:** Results of the pattern meanings test: mean values and standard deviations (in parentheses) of fluency, flexibility, total response time and response time per meaning.

	**Pattern 1**	**Pattern 2**	**Pattern 3**	**Pattern 4**	**Pattern 5**	**Pattern 6**	**Overall**
Fluency	3.38	2.75	3.09	3.10	2.54	2.65	2.92
	(1.83)	(1.61)	(1.80)	(2.00)	(1.75)	(1.38)	(1.76)
Flexibility	2.90	2.34	2.59	2.70	2.20	2.19	2.48
	(1.48)	(1.38)	(1.35)	(1.64)	(1.46)	(1.08)	(1.43)
Total response time in seconds	70.13 (60.81)	71.29 (63.79)	77.42 (60.90)	76.00 (73.85)	82.51 (78.60)	73.91 (62.83)	75.20 (55.99)
Response time per meaning in seconds	23.09 (20.31)	27.82 (21.35)	28.33 (22.89)	28.60 (28.56)	37.74 (38.13)	29.00 (22.29)	29.09 (26.55)

#### 6.3.3. Alternative Uses Task

Results can be found in Table 6. *Fluency* and *Flexibility* constitute the same measures as in the pattern meanings test, only for the number of alternative uses named for each given object. *Response times per object* and *response time per use* can also be found in the table.

**Table 6:** Results of the alternative uses task: mean values and standard deviations (in parentheses) of fluency, flexibility, total response time and response time per use.

	**Carpet**	**Newspaper**	**Cup**	**Dental floss**	**Toothbrush**	**Overall**
Fluency	3.20	4.73	4.09	3.32	3.46	3.76
	(2.10)	(2.69)	(2.56)	(2.05)	(2.13)	(2.39)
Flexibility	2.43	3.72	2.95	2.67	2.18	2.79
	(1.56)	(1.98)	(1.85)	(1.57)	(1.42)	(1.77)
Total response time in seconds	79.09 (65.66)	87.93 (84.09)	82.12 (70.19)	79.57 (76.75)	77.89 (76.80)	81.32 (74.83)
Response time per use in seconds	27.76 (25.61)	20.26 (18.96)	21.36 (16.18)	25.64 (20.18)	24.79 (27.49)	23.94 (22.20)

#### 6.3.4. Insight Tasks

Answers were rated for their correctness. Answers that included parts of the right solution received a score of 0.5. For the candle problem 51 participants (37%) had the right answer, 48 (35%) answered partly right, and 39 (28%) did not give the right answer to the problem. Four participants (3%) came up with the correct solution to the Jack and Jill weight problem, and one (1%) answered partly right.

#### 6.3.5. Reliability Analysis

For reliability analysis, Cronbach's alpha was calculated for *Fluency* in the pattern meanings test (α = 0.91), in the alternative uses task (α = 0.93) and for the number of key presses in the ambiguous figures task (α = 0.89). As all values reached satisfying levels, mean values were calculated for each task and used as an indicator for performance.

#### 6.3.6. Comparison of Measures

The so computed indicators of *Fluency* in both the pattern meaning test and the alternative uses task, as well as for performance in the ambiguous figures task were correlated with each other. A strong (according to Cohen, 1992) positive correlation was found between performance on the pattern meanings test and the alternative uses task (*r* = 0.77, *p* < 0.001). Correlations of Fluency values in the pattern meanings test (*r* = 0.32, *p* < 0.001) and in the alternative uses task (*r* = 0.37, *p* < 0.001) with the ambiguous figures task had a medium effect size.

**Figure 10:**
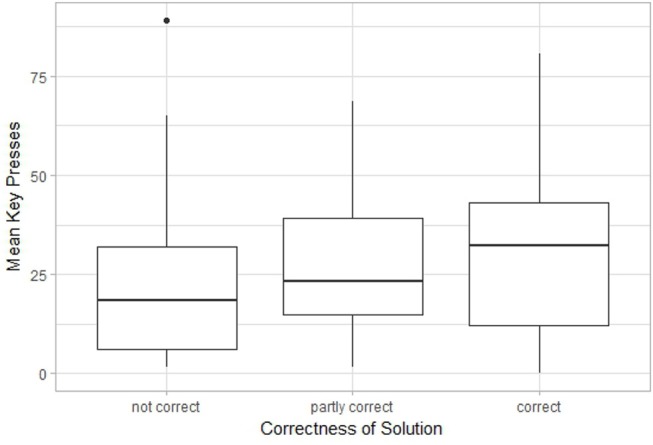
Boxplot comparing the participants' (i) answers to the candle problem (not correct, partly correct or correct), with (ii) their performance on the ambiguous figures task (measured as number of key presses).

**Figure 11:**
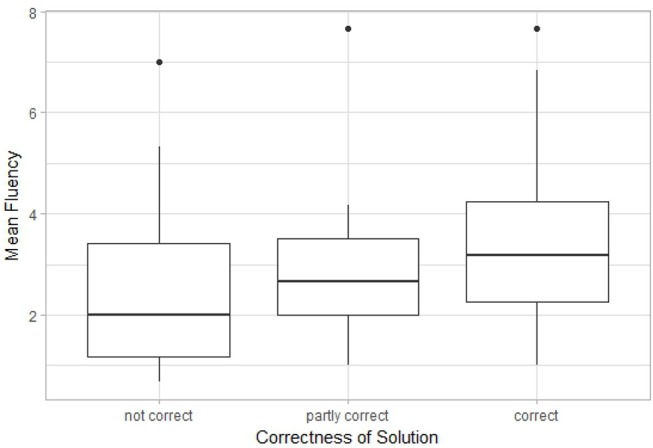
Boxplot comparing the participants that gave an answer to the candle problem that was not correct, partly correct or correct by their performance on the pattern meanings test, measured by *fluency*.

**Figure 12:**
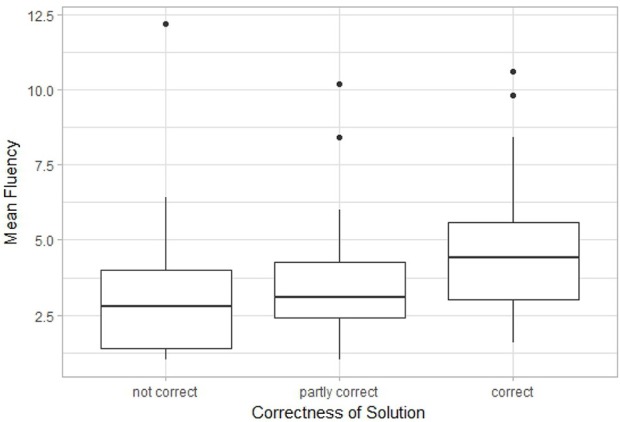
Boxplot comparing the participants that gave an answer to the candle problem that was not correct, partly correct or correct by their performance on the alternative uses task, measured by *fluency*.

A possible connection between performance on the insight problems and the other tasks was explored using the Kruskal-Wallis test by ranks. As only so few participants had the right answer to the Jack and Jill weight problem, only the answers to the candle problem were considered. Figures 10–12 show boxplots using performance on the candle problem as a grouping variable. These groups are compared by their performance on the ambiguous figures task, the pattern meanings test and the alternative uses task using the number of key presses and *Fluency* as indicators for performance. The Kruskal-Wallis test revealed differences in *Fluency* for the pattern meanings test (*Cohen's d* = 0.476, *p* < 0.05) and the alternative uses task (*Cohen's d* = 0.675, *p* < 0.001), but not for performance on the ambiguous figures task (*p* = 0.117, 1−β = 0.362). Pairwise comparisons revealed that for the pattern meanings test there was a significant difference for the comparison correct—not correct (*p* < 0.01). Additionally, there were significant differences for the alternative uses task; here, *Fluency* differed significantly between the groups correct—partly correct (*p* < 0.05), and correct—not correct (*p* < 0.001).

## 7. A Unified Model for Various Levels of Re-representation

In this section, all three levels of re-representation are shown in the context of a unified model.

**Feature Space Level**: At the feature space level, the input stimulus *I* is formally represented as a set of features:

$$\begin{array}{l} I=\left\{fg_{i}|i=1\text{ to }n\right\} \end{array}$$

where *n* = total number of individual features perceived *fg*_*i*_ = *ith* feature of the input stimulus. The re-representation here involves matching a subset of features of the input to known visual patterns; this type of re-representation involves grouping the features under various known patterns, searching and comparing them to patterns and objects in KB and then interpreting them as one of these known patterns or objects.

A) Ambiguous Figures test

In the context of ambiguous figures, re-representation happens as follows. Given the rabbit-duck problem in Figure 5, when the set of features *fg*_1_−*fg*_6_ are grouped together and *fg*_1_, *fg*_5_, *fg*_6_ are matched to the visual stimulus of *beak*, *feathers* and *backofhead*, respectively, the figure is represented as a *duck*. By contrast, when the features *fg*_1_, *fg*_5_, *fg*_6_ are matched to the visual stimulus of *ears*, *fur* and *pout*, the figure is represented as a rabbit. Re-representation in this case is the process of regrouping the initial sets of features in different ways and matching them to a different pattern.

**Figure 13:**
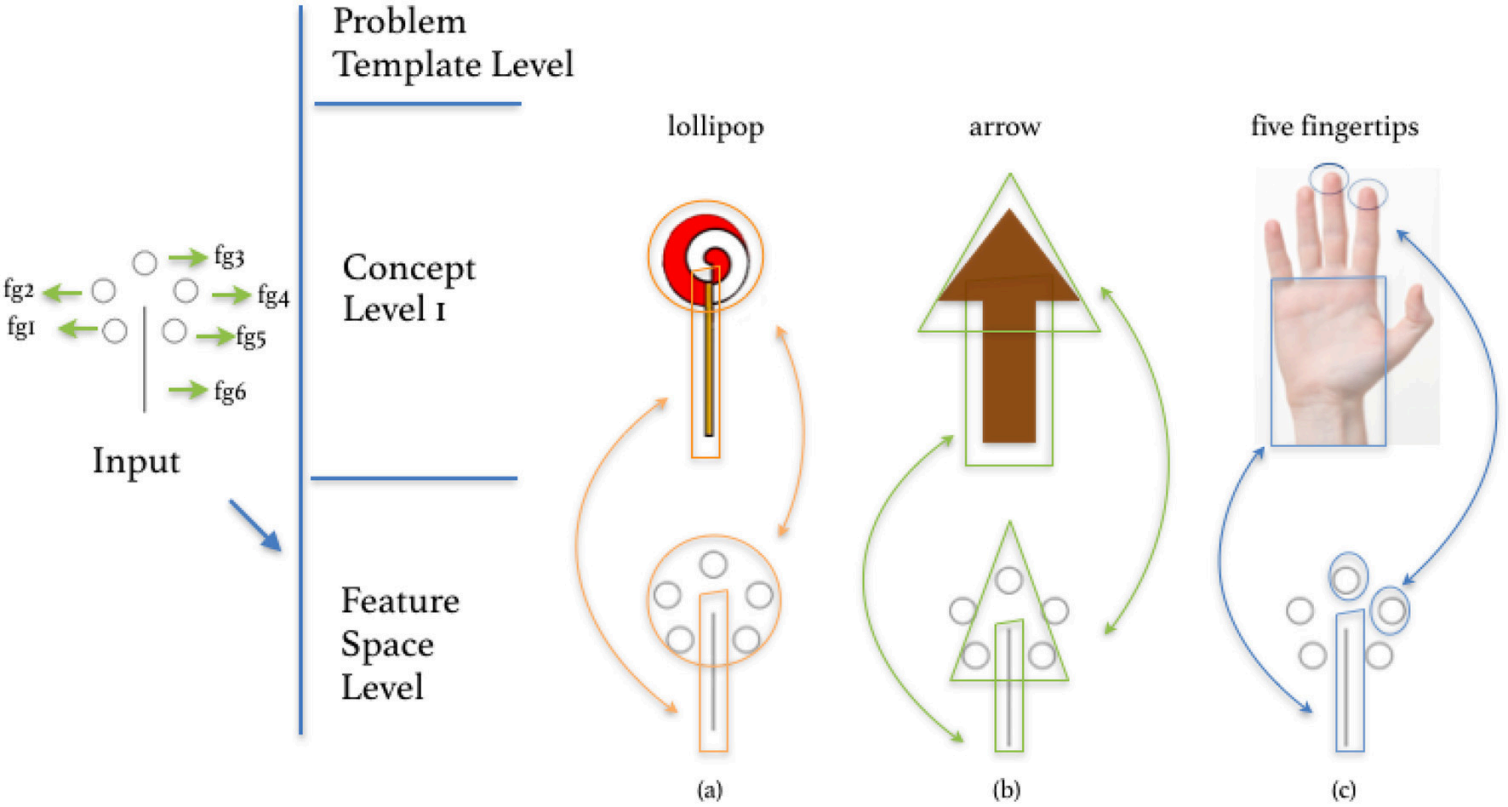
Features re-represented as different objects in the Pattern Meanings task. **(a)** lollipop, **(b)** arrow, and **(c)** five fingertips.

B) Pattern and line meaning testIn the context of the patterns meanings test (Figure 13), when the agent focuses on all features *fg*_1_−*fg*_6_, and *fg*_1_−*fg*_5_ are grouped together in the Gestalt of a circle, the pattern can be represented/interpreted as a lollipop (Figure 13a). When the agent selects and groups features *fg*_1_, *fg*_3_ and *fg*_5_ as the vertices of a triangle (Figure 13b), the set of features can be re-represented as an arrow. Similarly, when the roundness of *fg*_3_, *fg*_4_ is emphasized and they are matched to a known visual pattern of fingertips, the set of features *fg*_3_, *fg*_4_, and *fg*_6_ can be re-represented as five fingertips and palm (Figure 13c). The various re-representations takes place as follows:

$$\begin{array}{l} \begin{array}{c} I\to \left\{fg_{1},fg_{2},fg_{3},fg_{4},fg_{5},fg_{6}\right\}\to \left[\text{circular shape, stick}\right]\to object\left(\text{‘lollipop'}\right)\\ I\to \left\{fg_{1},fg_{3},fg_{5},fg_{6}\right\}\to \left[\text{three vertices, supporting line}\right]\to object\left(\text{‘arrow'}\right)\\ I\to \left\{fg_{3},fg_{4},fg_{6}\right\}\to \left[\text{two fingertips, palm}\right]\to object\left(\text{'hand'}\right) \end{array} \end{array}$$

It is important to mention that even when the same set of features are focused upon, there can be multiple re-representation outcomes. For example, in the same set of features are being focused upon in both (a) and (b); however in (a) the set of features are re-represented as a lollipop while in (b) as a tree. Re-representation in this case is the ability to regroup the features and re-map them to a different known pattern or object.

**Concept Level**: The object level of re-representation can happen at follows. In the CreaCogs framework (Olteţeanu and Falomir, 2016):

1. fs_shape refers to the feature space of shapes in the knowledge base (KB),
2. fs_material refers to the feature space of material in the KB,
3. fs_color refers to the feature space of colors in the KB,
4. fs_size refers to the feature space of height, depth and width of the objects in the KB,
5. fs_affordance refers to the feature space of actions/motions that can be done using the object,
6. fs_name refers to the feature space of object names in the KB .

An object/concept X in the CreaCogs framework is described as an activation of its features as follows:

$$\begin{array}{l} \;\forall X\exists s\;\in \;fs\_shape,\;\exists m\;\in \;fs\_material,\;\exists c\;\in \\ fs\_color,\;\exists z\;\in \;fs\_size,\;\exists a\;\in \;fs\_affordance,\;\exists n\;\in \;fs\_names\\ \;objext\left(x\right)\;\subseteq \;[shape(X,\;s)\;\wedge \;material(X,\;m)\;\wedge \\ color(X,\;c)\;\wedge \;size\;(x,z)\;\wedge \;affordance(X,a)\;\wedge \;name\;(X,\;n)] \end{array}$$

According to this scheme of formalization, the object cup and vase can be described as:

$$\begin{array}{l} object(cup)\to [shape(cup,high\_convexity)\wedge material(cup,glass)\wedge \\ \;affordance(cup,to\_drink\_from)\wedge name(cup,hcc\_cup)\\ object(vase)\to [shape(vase,high\_convexity)\wedge material(vase,ceramic)\wedge \\ \;affordance(vase,keep\_flowers\_in)\wedge name(vase,hcc\_vase) \end{array}$$

**Figure 14:**
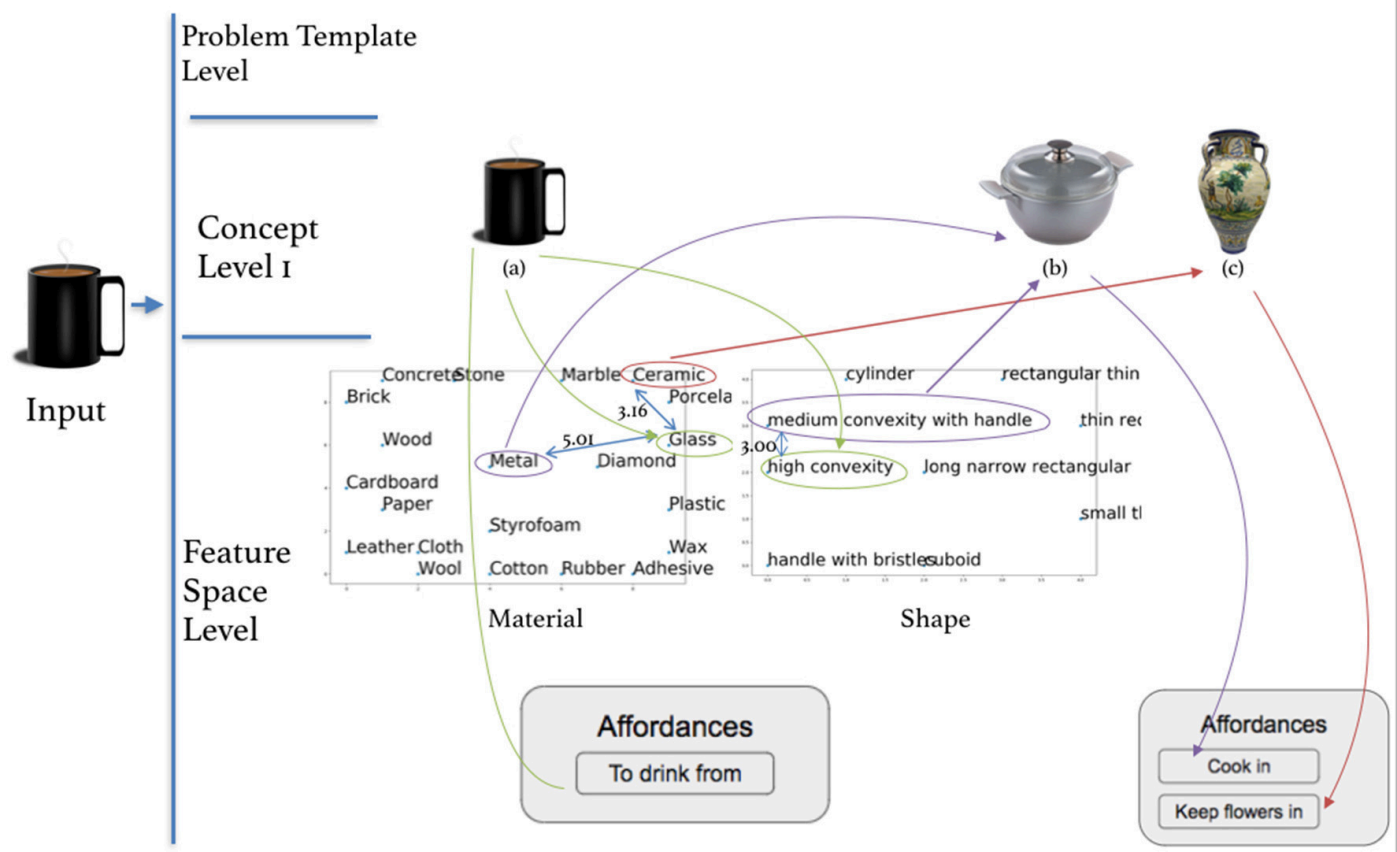
Objects and properties re-represented as different objects. **(a)** cup, **(b)** pot, and **(c)** vase.

The re-representation at this level is done by comparing the original object to the other object in terms of object properties such as material, shape, size etc. This is shown in Figure 14 and has been implemented computationally in a system that can come up with different uses for objects, by recasting them as objects which have similar properties (Olteţeanu and Falomir, 2016). In a more general form, assertions of the form *I can use object B to replace object A, in the context of task X* can be taken as *I can re-represent (a subset of) the features and properties of object A (which have been connected to task X) as object B*.

The OROC system (Olteţeanu and Falomir, 2016) establishes that the shape and material of a *pot* (“medium_convexity_with_handle”, “metal”) are similar to that of a *cup* (“high_convexity,” “glass”), and draws inferences about the affordances of a *pot* also applying to a *cup*. This is a form of re-representing a *pot* as a *cup*. Similarly, the features of a *cup* can be re-represented as a *vase*, by noticing the similarity between the shape and material of *vase* to that of *cup*. This object *cup* (as shown in Figure 14) thus has its constraints relaxed and inherits affordances from the object *vase*:

$$\begin{array}{l} object(cup)\to [shape(cup,high\_convexity)\wedge material(cup,glass)\\ \;\wedge affordance(cup,keep\_flowers\_in)\wedge name(cup,hcc\_cup) \end{array}$$

**Problem Template Level**: A problem template is formally represented as a set of concepts (*C*_*i*_), relations (*R*_*i*_), actions (*H*_*i*_), goals (*G*_*i*_) and constraints (*Cx*_*i*_). Where,

Concepts are everyday objects with properties such as shape, material, size etc.

Relations are an association between two or more concepts.

Actions are the action performed on the concepts which may or may not lead to a change in relation.

Goals are a set of concepts and relations.

Constraints describe the resource or action limits of the task.

For the two strings problem, the concepts, relations, goal and constraints can be conceptualized as follows:

- *C*_1_, *C*_2_−*String*_1_, *String*_2_
- *C*_3_−*Person*
- *C*_4_−*Chair*
- *C*_5_−*Pliers*
- *C*_6_−*Jar*_*of*_*nails*
- *C*_7_−*Sheets*_*of*_*paper*
- *C*_8_−*Nails*
- *R*_1_, *R*_2_−*hanging*(*String*_1_, *ceiling*), *hanging*(*String*_2_, *ceiling*)
- *R*_3_, *R*_4_, *R*_5_−*onFloor*(*Chair*), *onFloor*(*Sheets*_*of*_*paper*), *onFloor*(*Jar*_*of*_*nails*), *onFloor*(*nails*)
- *R*_6_−*holds*(*Person, String*_1_)
- *G*_*sol*_−{*tie*(*String*_1_, *String*_2_)}
- *Cx*_1_−if*R*_*x*_ = *holds*(*Person, String*_1_), then¬*Ry* = *reach*(*Person, String*_2_)

*C*_*problem*_ = {*C*_1_, *C*_2_, *C*_3_, *C*_4_, *C*_5_, *C*_6_, *C*_7_, *C*_8_}

*R*_*problem*_ = {*R*_1_, *R*_2_, *R*_3_, *R*_4_, *R*_5_, *R*_6_}

*H*_*problem*_ = {}

*G*_*problem*_ = {*G*_*sol*_}

*Cx*_*problem*_ = {*Cx*_1_}

*I* = {*C*_*problem*_, *R*_*problem*_, *H*_*problem*_, *G*_*problem*_, *Cx*_*problem*_}

**Figure 15:**
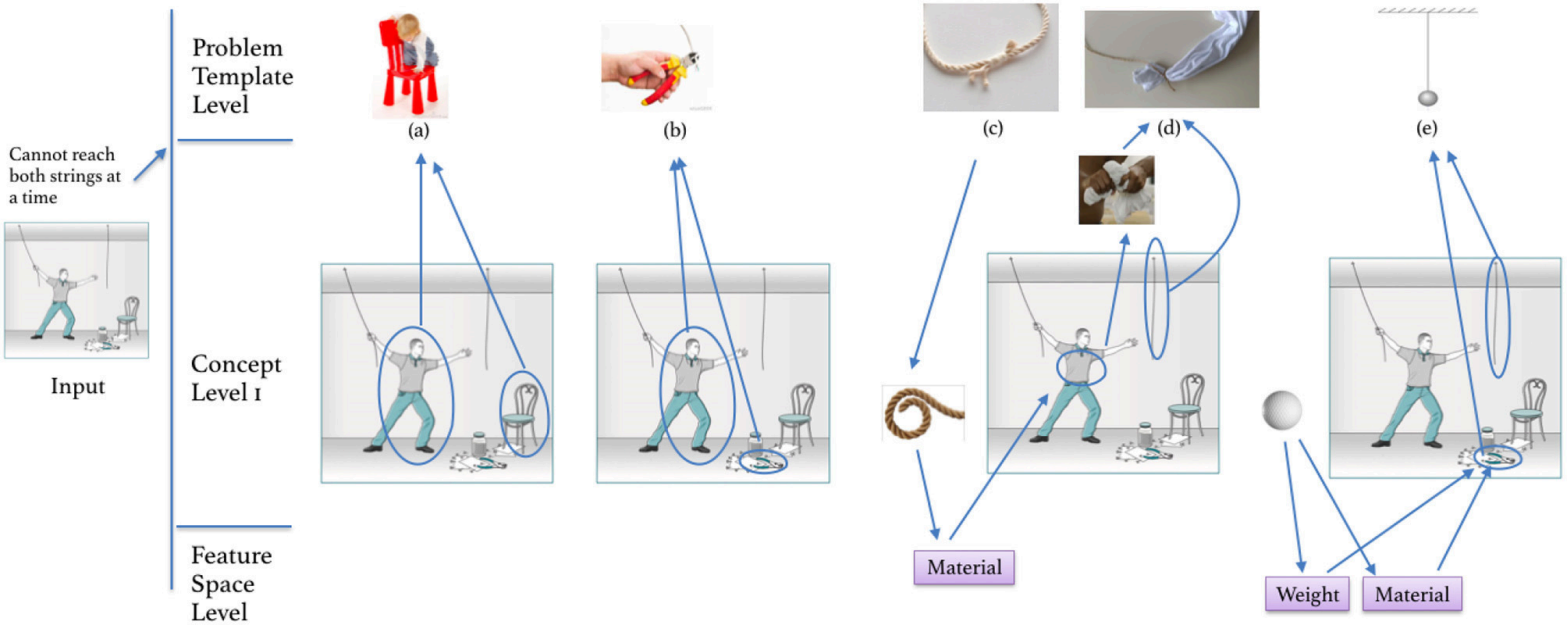
Objects and problem re-representation in the two strings problem. **(a)** climbing on chair template, **(b)** extending arm by using pliers, **(c)** tying two ropes, **(d)** tying rope and t-shirt, and **(e)** pendulum.

The re-representation here is of objects, objects sets and problem templates as different objects and problem templates. Figure 15 shows re-representation processes at this level in the context of the two strings problem. The of the problem is to tie the two strings together. The original problem template was the person trying to grab the second string while holding on to the first string. But the constraint specifically forbids this action, therefore other problem templates need to be triggered and applied to find a solution.

In Figure 15a the initial problem is re-represented through a template where the person is trying to hold the second string from a different height, assuming this will help reach further. The objects in the room (*chair*, *agent*) are thus re-represented as elements of this problem. In Figure 15b the template of trying to grab the other string using the pliers is triggered. This can be triggered either bottom up—from the object to the template level (by noticing the pliers exist in their room and thinking of their functionality), or top down, by thinking of ways to extend one's hand (the pliers would help reach a bit further).

Figures 15d,e show even more interesting cases of re-representation, as in them re-representation takes place at both problem template and concept level. In Figure 15d, the T-shirt of the man is re-represented as a rope. Then, the original problem is re-represented using a template of elongating the string by knotting two strings, where the template has been relaxed to tie the T-shirt to the rope. In Figure 15e, the pliers are re-represented as a weight, and then together with the string re-represented as a pendulum. This is probably triggered by and enables the use of a problem template containing knowledge about swinging the pendulum to get the rope to move toward you. In all the cases, the original problem is being re-represented with the help of different problem templates, and sometimes objects are re-represented as well.The re-representation at this level is formalized as

*I* → {*C*1, *C*2, *C*3} → PT(original):{*C* = {*C*_1_, *C*_2_, *C*_3_}, *R* = {*holds*(*C*_3_, *C*_1_)}, *G* = {*tie*(*C*_1_, *C*_2_)}}

*I* → {*C*_2_, *C*_3_} → PT(elongate_string):{*C* = {*C*_2_, *Tshirt*}, *H* = {*attach*(*C*_2_, *Tshirt*}, *G* = {*C*_*new*_ = {*longer*_*string*}}

*I* → {*C*_2_, *C*_5_} → PT(make_pendulum):{*C* = {*C*_2_, *C*_5_}, *R* = {*attached*{*C*_2_, *C*_5_}, *H* = {*swing*(*C*_2_)}, *G* = {*C*_*new*_ = {*pendulum*}}

## 8. Discussion

### 8.1. Discussion of the Results

The goal of this study was to investigate if there is an ability of re-representation that underlies performance on the ambiguous figures task, the pattern meaning test, the alternative uses test and insight problems. Indeed, many of the obtained results lead in that direction. The results of Wiseman et al. (2011) and Doherty and Mair (2012) could be confirmed by showing correlations between the ambiguous figures task and the alternative uses task, as well as between the ambiguous figures task and the pattern meanings test, with a much ampler participant size. Additionally, a correlation between the alternative uses task and the pattern meanings test was shown, with a much higher effect size.

A possible connection to performance on the insight tasks was explored using performance on the candle problem as a grouping variable. This was due to several reasons. First, due to the fact that in this study only two insight problems were given, it was not possible to explore the data at an interval level. Also, the Jack and Jill weight problem turned out to be quite difficult for participants, so that only very few were able to solve it. This limited the analysis to a group-wise comparison, dividing participants by their ability to provide a correct or at least partly correct solution for the candle problem. This was not possible for the Jack and Jill weight problem, as the groups would have been too different in their size. Due to violation of the assumption of normality, it was also not possible to conduct an ANOVA, thus analysis was limited to non-parametric tests. These revealed that on average, people with higher fluency ratings would have a higher chance at answering the candle problem correctly, indicating that these tasks share common features. This notion does not seem to hold true for the ambiguous figures task, though this comparison lacks power and ought to be investigated in future studies.

Generally our results support the hypothesis of an underlying ability of re-representation. The correlations between the ambiguous figures task, the pattern meanings task and the alternative uses test provide strong evidence for that notion, and insight task results, though exploratory in their nature, show some clear tendencies. What is however notable in the correlational results obtained is that they do not directly reflect the proposed level structure, as seen in Figure 4.

The correlation of the pattern meanings task with the alternative uses task shows a higher effect size than the other two correlations. From our theory, we would expect to see the ambiguous figures task and the pattern meanings task more closely related, as both involve the re-representation of features, in contrast to the alternative uses test, where participants need to re-represent objects. One thing that could explain this is the aspect of motivation. As there was no time limit given for the pattern meanings task and the alternative uses test, motivation might have played an important role in how long participants were trying to come up with new solutions. This was not the case for the ambiguous figures task, where participants had a time limit of 1 min after which the task was over, so that motivation might not have been as important.

The fact that in one task the possible representations were given, while in the other two participants had to come up with their own, might also have an influence on motivation and participant performance. Verbal fluency may also have an impact on the results, in that people who generally have a higher verbal fluency may be better at coming up with more answers in the pattern meanings task and alternative uses task. In future studies, the influence of these possible confounds should be investigated and, if necessary, controlled for.

### 8.2. General Discussion

Three levels of re-representation have thus been described, in the context of four empirical tasks. These tasks can be used to explore the relation between the different levels and compare computational models and artificial cognitive systems output to human output. A similar type of computational process could thus be used to solve all these types of tasks, with differences in terms of the type of knowledge and amount of knowledge used.

**Figure 16:**
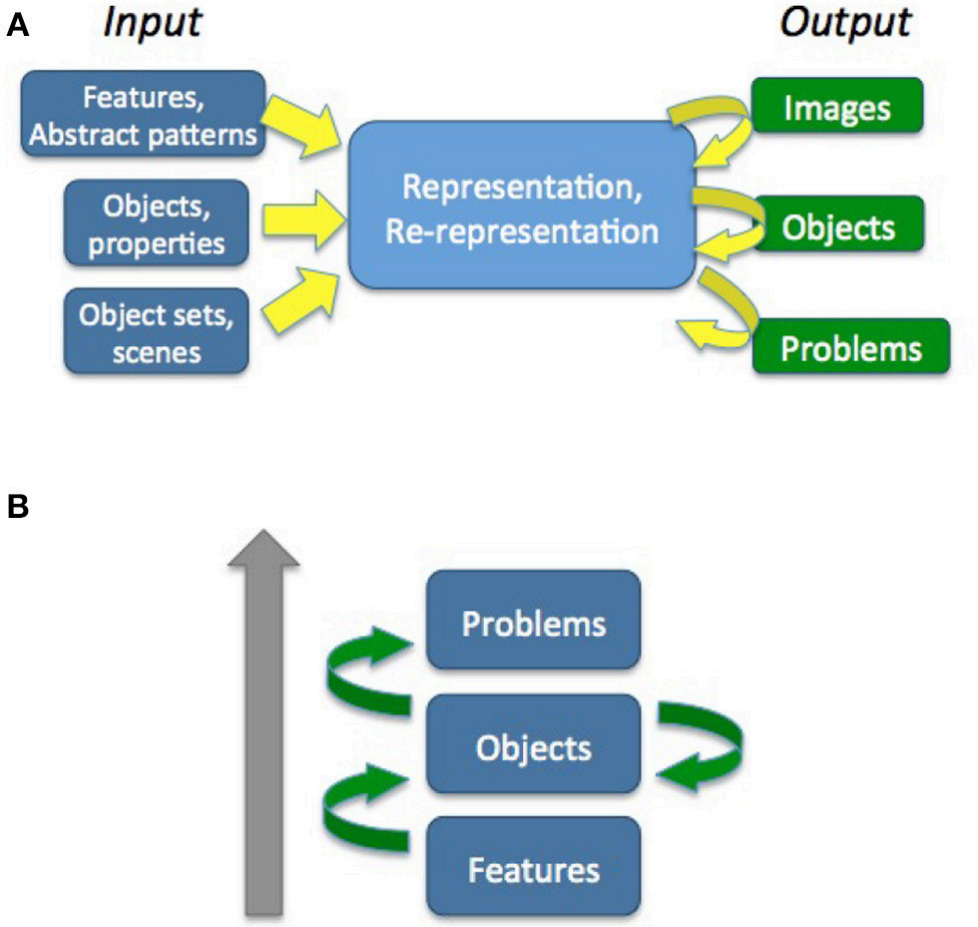
Re-representation. **(A)** As the same process, with different types of input and output; the output changes as a function of the process. **(B)** Re-representation (green arrows) acting at and between different levels of representational complexity.

At the computational system level, this requires an integrated multi-level modeling approach: a unified framework in which all three levels are modeled, and in which only the input, output, and access to the amount of memorized knowledge brought to the process changes, but the process stays the same or is similarly implemented. This is showcased in Figure 16A. At a coarse granularity, in the context of the levels previously analyzed, re-representation seems to be a process of *grouping various input features into different possible known outputs, which are already known* (or in structures similar to those outputs) *in order to obtain different functionality* of the representation, access different affordances connected to other objects, access different possible actions connected to other problem templates or action plans, or *transform the initial representation altogether, creating a new object, problem template* etc.

The output of the various levels which have been described here defines the interpretation or types of representations which are projected unto the input by the cognitive system, or the secondary properties which might follow from this representation (like new affordances, new solutions). Sometimes the output of a new representation is clear, and at other times this can only be observed because of its secondary properties.

As shown in Figure 16B, this approach posits that re-representation, displayed by the green arrows, acts between and at multiple levels of representational complexity. Integrative cognitive frameworks or architectures should thus be able to model, explain and display the process at all these levels. One of the inherent difficulties of modeling at all these levels will of course be one of representational complexity. Not to be neglected are also possible cascade effects of re-representation between multiple levels. For example, focusing on a different subset of features might make a solver see an object as a different potential object, which could qualify it for affordances and grouping in a different set of objects that together would make the problem be re-represented. Before accounting for such cascade effects, perhaps each possible level of re-representation should be studied in more depth on its own.

A computationally similar view on different processes will help simplify the modeling and implementation of each of these processes and tasks in computational cognitive systems. This will allow the further study of these tasks. If indeed the process is the same or similar at the three levels expressed here, then this unified synthetic approach should help understand re-representation in a much clearer manner by comparative exploration and experimentation of the specific levels, assuming initial differences in representation complexity (and in the manipulation of representations of different type and/or complexity). Association, similarity or other processes which might make re-representation possible have been tackled elsewhere (Olteţeanu, 2014; Olteţeanu and Falomir, 2015, 2016).

The current results of our study point in the direction of a relationship between the proposed tasks. However, no empirical argument in favor of a hierarchy of levels re-representations has yet been obtained, and one of our reviewers rightly points out that all these types of representations could co-exist in parallel. In future work we may be able to use a computational model to differentiate between these two hypotheses. We however believe that the three levels are useful artifacts for thinking about and modeling the types of information which is being processed: features, concepts and objects, problem templates (sets of objects, relations and actions).

In their paper, Öllinger et al. (2008) examine how mental set affects process of representational change. Mental set is defined as the tendency to solve certain problems in a fixed way based on previous solutions to similar problems. In CreaCogs, the corresponding element to mental set is fixation within a problem template which is triggered to solve a problem. Problem templates are structured sets of actions and ways of solving problems saved in the knowledge base (or memory of the solver). They are based on previous knowledge of objects, their relations, object properties, actions and how these have satisfied previous goals. Mental set is considered to be an artifact of the selection process (Anderson, 1982; Newell, 1994; Lovett and Anderson, 1996) In CreaCogs, the selection problem still stands, though it is not one we focus on this paper. That is, mental set can happen because one of the problem templates is selected preferentially over others, and the agent doing the problem solving is finding it difficult to re-represent the problem via a template which would provide more chances of success. One way to work on this problem in CreaCogs in the future would be to make assumptions or gather data on how familiar various templates are to various solvers, and then encode this computationally, through adding weights to the various templates.

In Öllinger et al.'s paper, representational change involves at least two possibilities—chunk decomposition and constraint relaxation. Chunk decomposition is analogous to object decomposition in CreaCogs, as an object which is perceived as a whole can be broken down to its constituent parts (or further sub components), and its components can be applied to solving the problem, or regrouped in other object configurations (the same stands for problem templates). The parallel to constraint relaxation in CreaCogs is relaxing the natural and learned biases against breaking objects, using problem templates in an already familiar way, crossing common sense or common practice norms or aesthetic values. This helps ease the re-representation process.

Öllinger et al. focus on the influence of various strategies, like chunk decomposition and constraint relaxation (or just standard type) on the participants further ability to solve problems requiring the same or other types of representational change. They however do not model nor describe the representational change process in terms which could serve for a computational implementation. The present paper does not make a statement regarding how representational change of one type influences other types of representational change. Instead, it focuses on finding various tasks with which the process of representational change could be explored and tested. This paper also aims for these empirical tasks to involve different types of stimuli, with different degrees of information complexity, and represent different knowledge levels of a cognitive agent and architecture. An initial re-representation mechanism which can be computationally modeled was put forward for all these information (and stimuli) levels.

The ambiguity of the various stimuli is also an interesting issue. In some cases, the ambiguity may allow for easier and more creative re-representations, and perhaps computational measures of ambiguity could be proposed at all the three levels. Our initial guess is that such a measure of ambiguity would require taking into account how many representations a certain scene can be parsed and represented as, which would probably be dependent on the cognitive system doing the re-representation. This would be hard to measure in natural cognitive systems, though perhaps a set of strong dominating possibilities of re-representations could be discerned for a particular set of stimuli, through empirical investigation. However, a computational measure of ambiguity would be interesting and much more feasible to deploy in artificial cognitive systems. The generativity of a particular artificial system might be a function of this measure of ambiguity and the system's capacity for re-representation.

This paper has put forward a multi-layered approach to re-representation. This approach aims to meaningfully integrate the performance in four different tasks to the study of re-representation as one cognitive process. If the differences based on knowledge between these different levels prove to influence the process itself in significant ways, re-representation can still be integrated as one class of processes. Such integration will support the unified empirical and computational study of a class of creative problem solving processes.

## 9. Conclusion and Future Work

This paper has proposed that re-representation, a key process in creative problem solving and human creativity, can and should be analyzed in a more integrative manner. Toward this, three levels of re-representation were explored. Tasks were used to exemplify and analyse the characteristics the re-representation process takes at each of these levels, in order to clarify the process itself.

An empirical study across these tasks confirmed our hypothesis that relations can be established between these tasks, replicated two previous studies with an ample number of participants and uncovered a new strong and significant relation between the Pattern Meanings Test and the Alternative Uses Test.

Our proposal was that the re-representation process be modeled all three levels: (i) re-representation of features as different objects or images, (ii) re-representations of objects and their properties as different objects, and (iii) re-representation of objects, objects sets, scenes and problems as different object and problem templates. An initial unified model for the re-representation process has been put forward and showcased at all these levels.

As future work we will aim to:

- Formalize each proposed level of re-representation;
- Attempt to model the various levels computationally under the same cognitive framework;
- Explore the underlying factors of empirical performance in these tasks.

## Ethics Statement

This study was carried out with written informed consent from all subjects. An approval of the ethics committee was not necessary, as no risks were attached to the study. All subjects gave written informed consent in accordance with the Declaration of Helsinki.

## Author Contributions

A-MO conceptualized and structured the theoretical foundation of the article. A-MO wrote sections 1–5 and the discussion in section 8, as well as the conclusion. A-MO and MS planned and deployed the empirical study. The data were analyzed by MS. MS wrote section 6. Post-revision, A-MO defined new research to be added to respond reviewers, and the unified model. AB wrote a new section 7 and added to the Discussion. MS did the Kruskal-Wallis analysis and added to the Discussion. A-MO supervised the process and revised and refined the new text.

### Conflict of Interest Statement

The authors declare that the research was conducted in the absence of any commercial or financial relationships that could be construed as a potential conflict of interest.

## Footnotes

^1^The difference between the line meanings test and the pattern meanings test is that the line is a continuous stimulus, and some set-ups assume it should not be broken into pieces by the participants when they are providing the interpretation.

^2^This level of re-representation could be discussed more widely in the context of concepts and their features, rather than objects. We chose a discussion on objects because in this case features are easier to determine, without running into the question how concepts and their properties are represented. This approach has the advantage that it can support the next level—that of insight problems—if the problems tackled are in the practical object domain.

^3^This can be thought of as familiarity with a particular graphical style of representation. For example, if one has never seen a rabbit represented as a 2D figure, in an iconic form and from the perspective from which it is usually represented, the initial interpretation of such a figure as a rabbit might be hindered.

^4^Other orders of process might also be possible. What we will focus on here is not the order, but on the coarser granularity of process at which various sets of features are represented and re-represented, or perceived and re-cast/seen as different objects.

^5^There is of course no bound which would restrict re-representation at the visual domain. Sticking to the visual however permits us to show figures of what we mean.

^6^The tongue or striker of a bell.

^7^Note: It is useful to emphasize that objects are in themselves groups: groups of object parts, and can also be seen as groups of properties, or even features.

^8^Because objects in various relations may have different affordances.

^9^*Like in the passage talking about this process with images, it is not the purpose of this paper to make an assumption about whether this process is top down, bottom up or an interaction between the two, but to showcase re-representation in general and the need for cognitive frameworks which integrate its multiple levels*.
