# Supplementary material for: Towards a Multi-level Exploration of Human and Computational Re-representation in Unified Cognitive Frameworks
Source: Front Psychol. 2019 Apr 30;10:940. doi: 10.3389/fpsyg.2019.00940 (PMC6502907; doi:10.3389/fpsyg.2019.00940)
Supplement: Supplementary file 1 [file Data_Sheet_1.pdf]

Supplementary Material:
Towards a Multi-level Exploration of Human and Computational Re-representation in Unified Cognitive Frameworks

1 SUPPLEMENTARY TABLES AND FIGURES

Table S1. Stimuli used in the ambiguous figures task

Picture 1	Picture 2	Picture 3
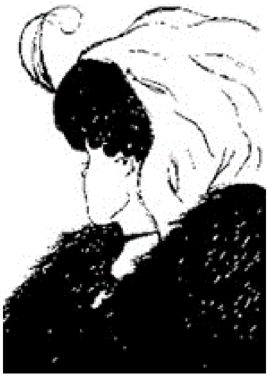	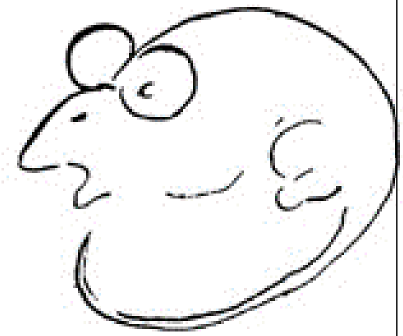	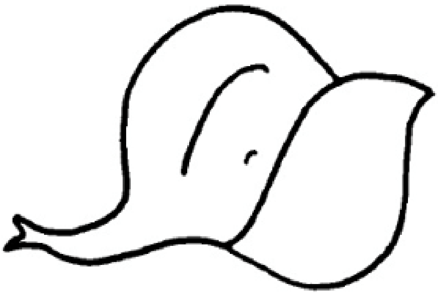
**Picture 4**	**Picture 5**	
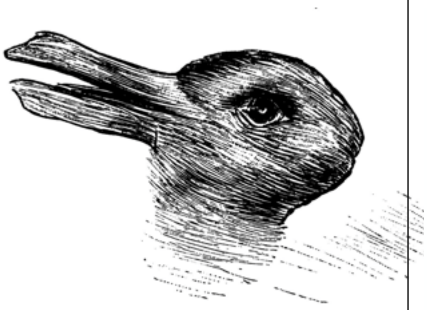	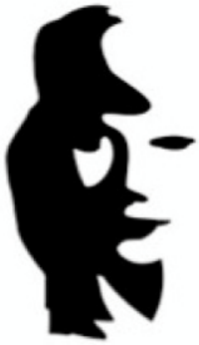	

Table S2. Stimuli used in the pattern meanings test

**Pattern 1** 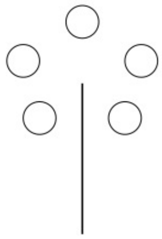	**Pattern 2** 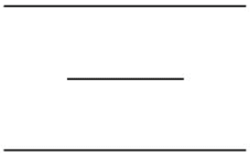	**Pattern 3** 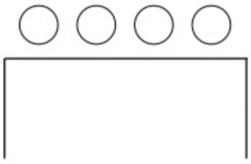
**Pattern 4** 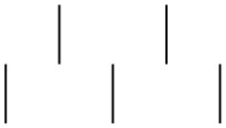	**Pattern 5** 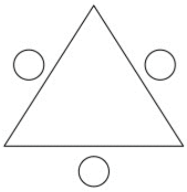	**Pattern 6** 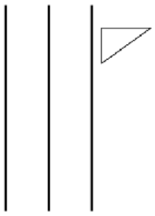

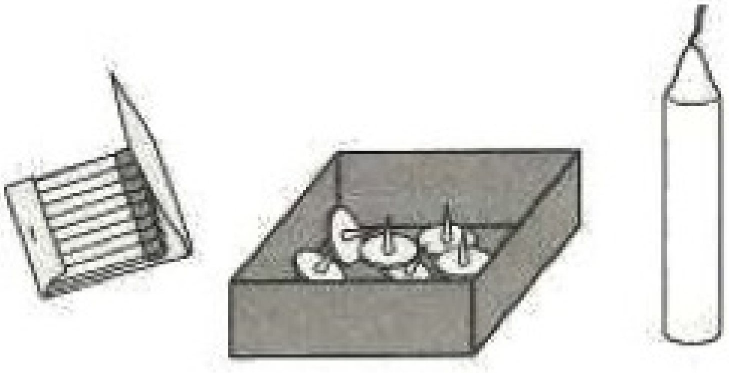

Figure S1. The candle problem

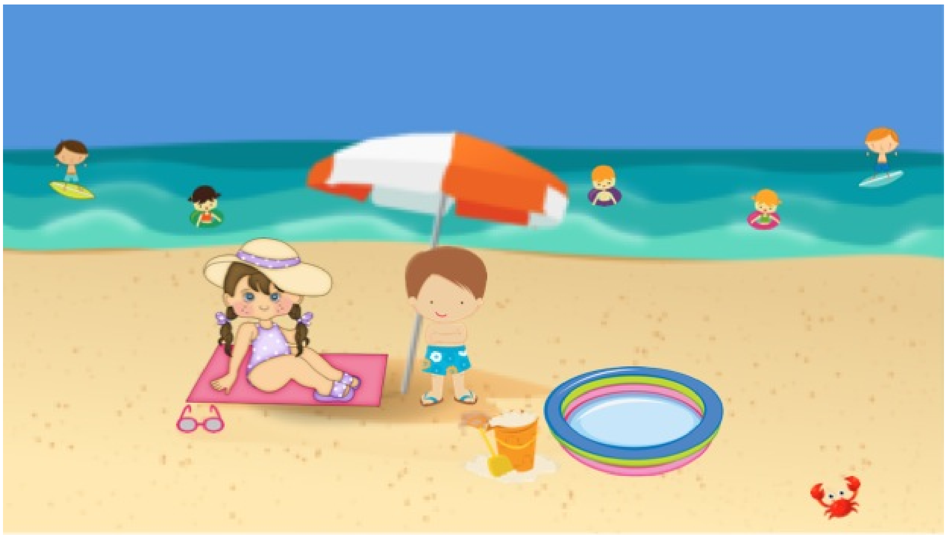

Figure S2. The Jack and Jill weight problem
